# Diabetes and Prediabetes in Children With Cystic Fibrosis: A Systematic Review of the Literature and Recommendations of the Italian Society for Pediatric Endocrinology and Diabetes (ISPED)

**DOI:** 10.3389/fendo.2021.673539

**Published:** 2021-04-29

**Authors:** Enza Mozzillo, Roberto Franceschi, Claudia Piona, Stefano Passanisi, Alberto Casertano, Dorina Pjetraj, Giulio Maltoni, Valeria Calcaterra, Vittoria Cauvin, Valentino Cherubini, Giuseppe D’Annunzio, Adriana Franzese, Anna Paola Frongia, Fortunato Lombardo, Donatella Lo Presti, Maria Cristina Matteoli, Elvira Piccinno, Barbara Predieri, Ivana Rabbone, Andrea Enzo Scaramuzza, Sonia Toni, Stefano Zucchini, Claudio Maffeis, Riccardo Schiaffini

**Affiliations:** ^1^ Department of Translational Medical Science, Section of Pediatrics, Regional Center of Pediatric Diabetes, Federico II University of Naples, Naples, Italy; ^2^ Pediatric Unit, S. Chiara Hospital, Trento, Italy; ^3^ Regional Center for Pediatric Diabetes, University of Verona, Verona, Italy; ^4^ Department of Human Pathology in Adult and Developmental Age, University of Messina, Messina, Italy; ^5^ SOD Pediatric Diabetology, Department of Women’s and Children’s, “G. Salesi” Children’s Hospital, AOU Ospedali Riuniti, Ancona, Italy; ^6^ Department of Woman, Child and Urological Diseases, S. Orsola-Malpighi University Hospital, Bologna, Italy; ^7^ University of Pavia, Pavia and Department of Pediatrics, "Vittore Buzzi" Children’s Hospital, Milano, Italy; ^8^ IRCCS Istituto Giannina Gaslini, Genova, Italy; ^9^ Diabetes Unit, Ospedale Brotzu, Cagliari, Italy; ^10^ Centro di Riferimento Regionale di Diabetologia Pediatrica A.O.U. Policlinico G. Rodolico, Catania, Italy; ^11^ Diabetes Unit, Bambino Gesù Children’s Hospital, Rome, Italy; ^12^ D.A.I. Pediatria, Ospedale Pediatrico Giovanni XXIII, Bari, Italy; ^13^ Department of Medical and Surgical Sciences of the Mother, Children and Adults - Pediatric Unit, University of Modena and Reggio Emilia, Modena, Italy; ^14^ Division of Pediatrics, Department of Health Science, University of Piemonte Orientale, Novara, Italy; ^15^ Diabetes & Nutrition Unit, Pediatrics, ASST Cremona, Cremona, Italy; ^16^ Pediatric Diabetology Unit, Meyer Children Hospital, Florence, Italy

**Keywords:** cystic fibrosis-related diabetes, prediabetes, oral glucose tolerance test (OGTT), continuous glucose monitoring, abnormal glucose tolerance, systematic review, recommendations, glargine insulin

## Abstract

Cystic fibrosis related diabetes (CFRD) is a comorbidity of cystic fibrosis (CF) that negatively impacts on its clinical course. Prediabetes is an important predictor of either CFRD development and unfavorable prognosis of CF in both pediatric and adult patients. International guidelines recommend insulin only in case of CFRD diagnosis. Whether early detection and treatment of prediabetes may contribute to improve the clinical course of CF is still debated. A subgroup of pediatric diabetologists of the Italian Society for Pediatric Endocrinology and Diabetology (ISPED) performed a systematic review of the literature based on predefined outcomes: impact of pre-diabetes on clinical outcomes and on the risk of developing CFRD; diagnosis of diabetes and pre-diabetes under 10 years of age; effectiveness of therapy on glycemic control, impact of therapy on pulmonary function and nutritional status. Thirty-one papers were selected for the analysis data presented in these papers were reported in tables sorted by outcomes, including comprehensive evidence grading according to the GRADE approach. Following the grading of the quality of the evidence, the entire ISPED diabetes study group achieved consensus for the Italian recommendations based on both evidence and clinical experience. We concluded that in patients with CF, prediabetes should be carefully considered as it can evolve into CFRD. In patients with CF and prediabetic conditions, after complete evaluation of the OGTT trend, glucometrics, glycemic values measured during pulmonary exacerbations and/or steroid therapy, early initiation of insulin therapy could have beneficial effects on clinical outcomes of patients with CF and prediabetes.

## Introduction

Improving survival in cystic fibrosis (CF) has caused an increase of the prevalence of comorbidities, with cystic fibrosis-related diabetes (CFRD) being the most common and affecting at least half of the adult CF population (1). CFRD has a negative impact on the clinical course of the disease increasing its mortality (2). In recent years, several lines of evidence have demonstrated that pulmonary function, microbiological colonization and nutritional status start to worsen several years prior to the diagnosis of CFRD (3). Early detection of pre-diabetes, defined in CF patients by the presence of abnormal glucose tolerance (AGT), impaired glucose tolerance (IGT), impaired fasting glucose (IFG) or indeterminate glycaemia (INDET) is therefore essential, although to date few studies have focused on pre-diabetes and its negative significant impact on the course of CF

The main cause of CFRD is insulin insufficiency with insulin secretion being increasingly impaired in correlation with exacerbation of pre-diabetes (4). According to this pathophysiological evidence, current guidelines of the International Society for Pediatric and Adolescent Diabetes (5) recommend insulin therapy initiation for the treatment of CFRD. However, no specific insulin therapies appear to have significantly distinct advantages both for an effective treatment of hyperglycemia and for their possible positive impact on the clinical course of CF (6). In addition, whether CF patients diagnosed with pre-diabetes could benefit from ‘early’ initiation of insulin therapy it is still debated (7).

The screening and diagnosis of CFRD and pre-diabetic conditions, as long as the effectiveness of the therapy for the treatment of CFRD and pre-diabetes represent two topics of major interest in the field of diabetes and CF. For this reason, a subgroup of pediatric diabetologists of Italian Society for Pediatric Endocrinology and Diabetology (ISPED) performed a systematic review of the literature and Grading of Recommendations, Assessment, Development and Evaluation (GRADE) profiles focusing on the above mentioned debated topics.

## Methods

A systematic review based on pre-defined outcomes for each question has been performed. The entire diabetes study group of ISPED compiled evidence profiles and achieved consensus for the final recommendations.

In regards to screening and diagnosis of diabetes and pre-diabetes in CF patients, the outcomes were: 1) impact of pre-diabetes on clinical outcomes (pulmonary function, number of pulmonary exacerbations and nutritional status) and 2) on the risk of developing CFRD; 3) diagnosis of diabetes and pre-diabetes in children under 10 years of age.

In regards to effectiveness of therapy in CFRD and pre-diabetes the outcomes were: 1) effectiveness of therapy on biochemical measures of glycemic control, as glycosylated hemoglobin (HbA1c), fasting and 2 h post-meal serum blood glucose values, and data derived from CGM download; 2) effectiveness of therapy in improving pulmonary function, 3) effectiveness of therapy in improving nutritional status.

Pulmonary function was analyzed in terms of forced expiratory volume in the 1st second (FEV1) and forced vital capacity (FVC). Pulmonary exacerbations were analyzed considering the following criteria: increased sputum volume, more frequent coughing, increased dyspnea, weight loss, change in the chest physical examination, absence from school or work because of illness, requiring hospitalization and antibiotic therapy. Pathogens colonization was considered on the results of microbiological investigations on deep pharyngeal aspirates. Pathogens colonization on deep pharyngeal aspirates was considered on the results of microbiological investigations. Nutritional status was evaluated as body mass index (BMI) and standard deviation scores (SDS) of weight and height.

The method used to perform this systematic review was based on the PICOS model (Population, Intervention, Comparison, Results, Study design). Inclusion criteria of studies are listed in **Table 1**. Exclusion criteria were studies not meeting the established outcomes and studies with animals. No restrictions were applied regarding the published paper’s language and patients’ age. The articles selected for this literature review include all those published from 1/01/2006 to 30/10/2020. The keywords used, also called “mesh” (MEdical Subject Headings) on PubMed, are the following: “diabetes diagnostic test AND cystic fibrosis,” “cystic fibrosis AND diabetes management,” “cystic fibrosis AND AGT,” “cystic fibrosis AND IFG,” “cystic fibrosis AND IGT,” “cystic fibrosis AND INDET,” “cystic fibrosis AND diabetes.” Systematic searches, using relevant keywords and search strings, were conducted on electronic databases (PubMed, Scopus, Google Scholar, CINAHL, Nursing reference center, Up to date and PsycINFO, Embase, CENTRAL) and clinical trial registers (http://clinicaltrials.gov; www.controlled-trials.com).

**Table 1:** PICOS model (population, intervention, comparison, results, study design) adopted in the systematic review.

Population	Patients with CF
Intervention	Diagnostic test and treatment of pre-diabetes and/or diabetes in CF
Comparison	Patients with CF not screened or not treated for pre-diabetes or diabetes
Results	Screening and diagnosis of diabetes and pre-diabetes -Impact of pre-diabetes on the clinical course of CF - Pre-diabetes and risk to develop CFRD -Diagnosis of diabetes and pre-diabetes in patients with CF under 10 years of age Effectiveness of therapy in CFRD and pre-diabetes -Biochemical measures of glycemic control -Pulmonary function and number of pulmonary exacerbations -Assessment of nutritional status
Study design	RCTs, observational studies, prospective studies, cross-sectional studies, exploratory studies, case series, case reports, mix of qualitative and quantitative studies

In order to derive recommendations, a GRADE approach to rank the quality of a body of evidence was applied as reported in **Table 2** (8, 9). The final assessment of the quality of evidence was discussed and established by the entire subgroup.

**Table 2:** GRADE approach to ranking the quality of a body of evidence.

**High**	=	Further research is very unlikely to change confidence in the estimate of effect.
**Moderate**	=	Further research is likely to have an important impact on confidence in the estimate of effect and may change the estimate.
**Low**	=	Further research is very likely to have an important impact on confidence in the estimate of effect and is likely to change the estimate.
**Very low**	=	Any estimate of effect is very uncertain.

For each risk of bias (i.e. imprecision, inconsistency, indirectness, and publication bias), the authors had the option of decreasing their level of certainty one or two levels (e.g., from high to moderate). Since GRADE cannot be implemented mechanically, there is by necessity a considerable amount of subjectivity in each decision.

Following the assessment of the quality of the evidence, an assessment of the strength of the recommendation was made. A consensus for the final recommendations was achieved from the entire diabetes study group of the ISPED, and summarized in **Table 3**. According to the GRADE method, the strength of each recommendation is classified in four mutually exclusive categories: “strong” and “weak or conditional” in favor (positive) or against (negative) the use of a specific intervention, as reported in **Table 4** (10).

**Table 3:** Factors than can reduce or increase the quality of evidence.

Factors that can reduce the quality of the evidence	
Factor	Consequence
Limitations in study design or execution (risk of bias)	**↓** 1 or 2 levels
Inconsistency of results	**↓** 1 or 2 levels
Indirectness of evidence	**↓** 1 or 2 levels
Imprecision	**↓** 1 or 2 levels
Publication bias	**↓** 1 or 2 levels
**Factors that can increase the quality of the evidence**	
Factor	Consequence
Large magnitude of effect	**↑** 1 or 2 levels
All plausible confounding would reduce the demonstrated effect or increase the effect if no effect was observed	**↑** 1 level
Dose-response gradient	**↑** 1 level

**Table 4:** Assessment of the strength of a recommendation.

Strength of recommendation	Rationale
**Strong**	The panel is confident that the desirable effects of adherence to the recommendation outweigh the undesirable effects
**Weak or** **conditional** (depending on patient values, resources available or setting) **discretionary** (based on opinion of patient or practitioner) **qualified** (by an explanation regarding the issues which would lead to different decisions).	The panel concludes that the desirable effects of adherence to a recommendation probably outweigh the undesirable effects. However: - the recommendation is only applicable to a specific group, population or setting or - new evidence may result in changing the balance of risk to benefit or - the benefits may not warrant the cost or resource requirements in all settings
**No recommendation possible**	Further research is required before any recommendation can be made

We used these standard expressions and if sufficient evidence was not available recommendations were based on the panel opinion according to the current daily clinical practice.

## Results

**Figure 1:**
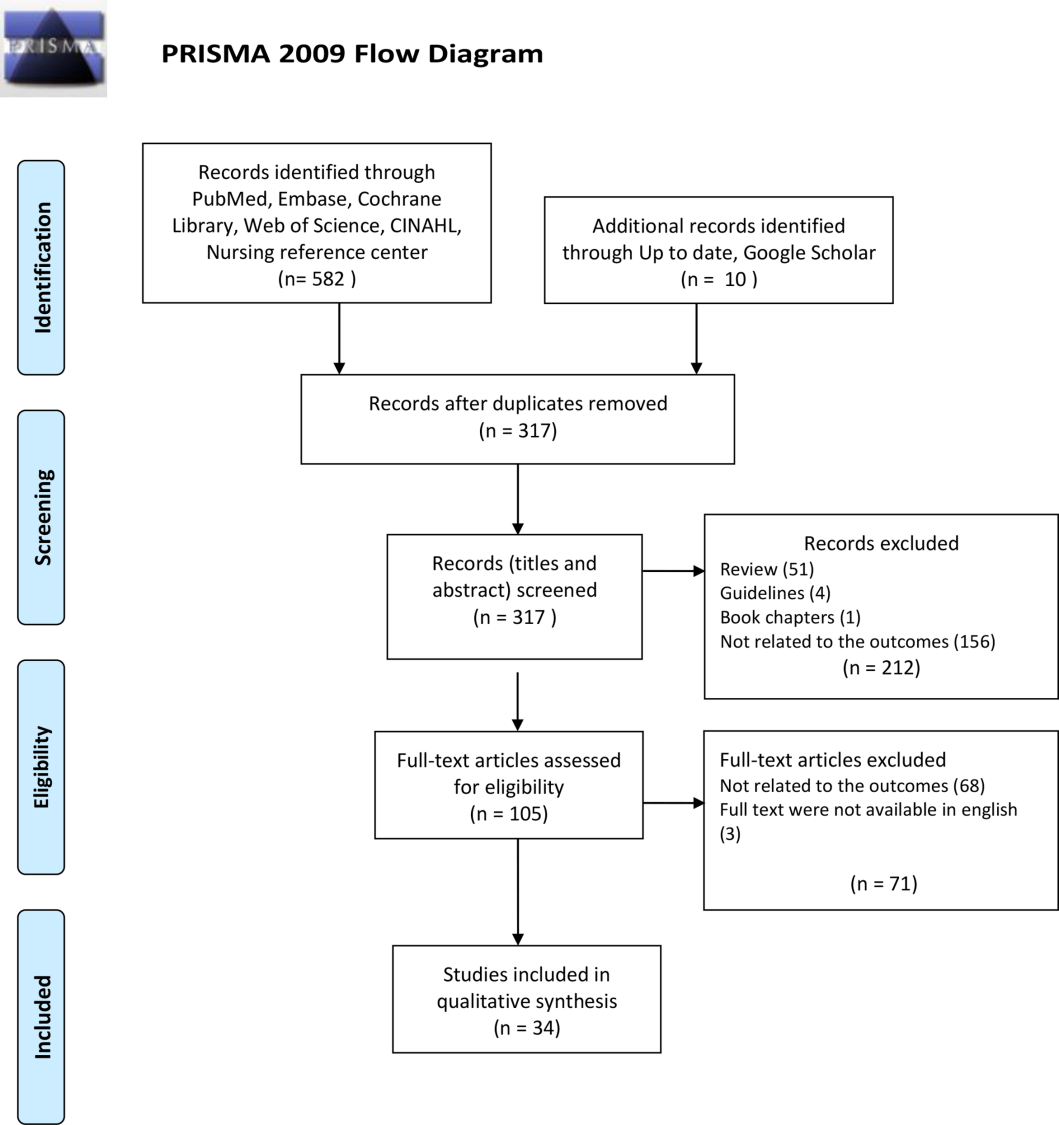
PRISMA Flow-Diagram.

After a careful evaluation of all databases available, 592 papers were found, 317 of whom were removed because they were duplicates (**Figure 1**). Among the 105 papers left, after reading the full text of each of them, only 34 papers were selected for the analysis because all the inclusion criteria were met.

Data from the 34 studies selected, including comprehensive evidence grading, are presented in **Tables 5**–**10**, sorted by outcomes.

**Table 5:** Impact of prediabetes on the clinical outcomes in CF patients.

Reference/Study	Study design Follow up period	Participants and Diagnostic Test	Pulmonary Function	Growth and Nutritional status	Pulmonary exacerbations and microbiological colonizations	Rating upgrade/downgrade	Evidence level
Banavath et al. (11)	Single-center observational case-control study Period of data collection: 2006 – 2016.	25 children tested with OGTT and divided in two groups: - Group A: 16 children with AGT (including IFG, INDET, IGT, and CFRD) - Group B: 9 children with NGT	Significant lower FEV1/FVC in children with AGT (p< 0.0001)	Significant lower BMI Z-score in children with AGT (p< 0.0001)	Significant higher rate of colonization by *Pseudomonas aeruginosa* and number of hospitalization in children with AGT (p<0.0001)	Downgrade (imprecision)	Moderate
Lavie et al. (12)	Single-center observational case-control study Period of data collection: not declared.	51 adolescents and adults tested with OGTT and divided in two groups: - Group A: 38 subjects with NGT - Group B: 13 subjects with IGT	Significant lower FEV1 in subjects with IGT (p= 0.014); significant negative correlation between FEV1 and fasting, 30 min, 1-h, 2-h and peak glucose measured during OGTT.	Significant lower BMI-SDS in subjects with IGT (p< 0.001)	Not evaluated	Downgrade (imprecision)	Moderate
Coriati et al., (13)	Single-center observational case-control study. Period of data collection: 2014-2016.	252 adults tested with OGTT categorized according their glucose tolerance status: - 99 subjects with NGT - 66 subjects with IGT - 45 subjects with INDET - 42 subjects with *De novo* CFRD	Significant trend of worsening of FEV1% across glucose tolerance categories (p= 0.038); CFRD and INDET subjects have comparable decreased FEV1% (p=0.996)	No significant trend of weight and BMI across glucose tolerance categories (p=0.723 and p=0.813 respectively)	Not evaluated	No	Moderate
Terliesner et al. (14)	Single-center retrospective observational case control study. Period of data collection: 1990 – 2014; mean follow-up duration of 13 years	16 adolescents who developed CFRD during the follow-up period compared to 16 age and gender-matched subjects who not developed CFRD.	Significant lower FEV1% and FVC% both at baseline and during Follow-up in subjects with CFRD	Significant lower Height and weight SDS% both at baseline and during Follow-up in subjects with CFRD, no significant differences in BMI and BMI-SDS.	Not evaluated	No	Moderate
Tommerdahl et al. (15)	Single-center observational study. Period of data collection: not declared. Follow-up duration: unavailable	52 CF patients (11 with NGT, 33 with AGT, and 8 with CFRD) aged 10-18 year were tested with OGTT. Three alternative criteria were considered: curve shape (biphasic vs. monophasic), time to glucose peak (≤30 min vs. > 30 min), 3. 1-h glucose (< 155 mg/dl vs. ≥155 mg/dl).	There were no differences between groups (biphasic vs. monophasic, ≤30 min vs. > 30 min, < 155 mg/dl vs. ≥155 mg/dl) in FEV%1 or FVC%	BMI z-score was significantly higher in the peak glucose at > 30 min group vs. the peak glucose at ≤30 min group. No other differences were found.	Not evaluated	Downgrade (risk of bias)	Low
Leclercq et al. (16)	Single-center observational case-control study. Period of data collection: 2009 – 2012.	38 children aged >10 years with NGT at OGTT tested with 3-day CGM: - Group A: 26 subjects with BG max <200 mg/dl (11 mmol/l); - Group B: 12 subjects with BG max > 200 mg/dl (11 mmo/l)	Significant lower FEV1% and FVC% in Group B (p=0.01 and p= 0.021, respectively)	No significant difference in BMI SDS between the 2 groups (p = 0.079)	Significant higher rate of colonization by *Pseudomonas Aeruginosa* in Group B (p=0.024)	Downgrade (imprecision)	Moderate
Olszowiec-Chlebna et al. (17)	Single-center retrospective observational study Period of data collection: 1996 -2009; mean follow-up duration of 8 years.	61 children tested with OGTT	Impaired glucose tolerance status was a significant risk factor for decline in FEV1% in children older than 10 years of age (p= 0.027)	Not evaluated	Not evaluated	Upgrade (Large magnitude of effect)	Moderate
Milla et al. (18)	Single-center prospective longitudinal observational study. Period of data collection: not declared; mean follow-up duration of 4 years.	187 children and 65 adults tested with OGTT divided according to their glucose tolerance status in three groups: - 69 subjects with NGT - 59 subjects with IGT - 24 subjects with CFRD	A significant decline in FEV1% and FVC% during the follow up period was observed in CFRD and IGT subjects, and not in NGT subjects	No significant differences in BMI trends across the glucose tolerance groups.	Not evaluated	No	Moderate
Bizzarri et al. (19)	Single-center prospective longitudinal observational study. Period of data collection: not declared; mean follow-up duration of 3 years.	Children tested with OGTT during puberty divided according to their glucose tolerance status in three groups: - Group A: 52 subjects with NGT - Group B: 17 subjects with CFRD	Significant lower FEV1% and FVC% in Group B A both at baseline and during follow-up period (p = 0.01 and p = 0.02, respectively)	Significant lower BMI-SDS in Group B both at baseline and during follow-up period (p = 0.01 and p = 0.04, respectively).	No significant differences in the rate of colonization by *Pseudomonas Aeruginosa* in Group B both at baseline and during follow-up period (p= 0.78 and p=0.38, respectively); Significant higher rate of hospitalizations and outpatient clinic visits during the follow-up in Group B (p= 0.003 and p=0.01, respectively).	No	Moderate
Van Sambeek et al. (20)	Single- center retrospective case-control study. Period of data collection: 2012 to 2013.	88 children and adults tested with OGTT and categorized according their glucose tolerance status in three groups: NGT, CFRD and IGT.	Significant lower FEV1% and FVC% in CFRD subjects with HbA1c level > 6.5% compared to CFRD subjects with HbA1c level < 6.5%	Not evaluated	Significant higher rate of pulmonary exacerbation in CFRD subjects	No	Moderate
Limoli et al. (21)	Single center observational case-control study. Period of data collection: 2012 or 2013.	91 children and 134 adults tested with OGTT categorized according to their glucose tolerance status in two groups: - 136 subjects with NGT - 89 subjects with CFRD	Significant lower FEV1% in CFRD subjects	Not evaluated	Significant higher rate of *P. aeruginosa* and *S. aureus* confections and pulmonary exacerbations requiring IV antibiotics in CFRD subjects.	No	Moderate
Reece et al. (22)	Multicenter retrospective observational case control study. Period of data collection: 2013.	749 children and adults categorized in 6 groups depending on their colonization status for *Pseudomonas aeruginosa* and *Aspergillus fumigatus*	Not evaluated	Not evaluated	Patients persistently colonized with PA had a higher prevalence of CFRD diagnosis (p = 0.012).	No	Low
Merlo et al. (23)	Multicenter longitudinal observational study Period of data collection: 1998 – 2002, follow-up duration of 5 years.	4293 children and adults in which data about demographics, anthropometrics, spirometry, respiratory culture results, comorbidities, antibiotic usage, and hospitalizations were collected.	Not evaluated	Not evaluated	CFRD was a significant risk factor for the acquisition of multiple antibiotic resistant P. aeruginosa infection (hazard ratio [HR], 1.64; 95% confidence interval [CI], 1.11 to 2.43).	No	Elevated

**Table 6:** Impact of prediabetes on the risk to develop CFRD.

References	Study design Follow up duration	Participants	Main outcomes	Rating upgrade/downgrade	Evidence level
Larson Ode K et al., 2010	Single-center retrospective study. Follow-up: 5 years.	62 CF children aged 6-9 years tested with OGTT. Glucose tolerance categories: AGT (50%) and NGT (50%)	Odds of developing diabetes were 11 times greater AGT patients (p<0.001)	No	Moderate
Schmid et al. ( (24)	Multicenter longitudinal study. Mean follow-up: 3.6 ± 2.1 years.	1093 CF patients aged 10 years or older with at least two valid OGTTs each and no CFRD at their first OGTT. Glucose tolerance categories groups: NGT (76.7%), IFG (6.4%), IGT without IFG (14.2%), IGT with IFG (2.7%). INDET (269 patients) and no-INDET (269 patients) only for a subgroup of patients)	Incidence of CFRD was significantly higher in the IFG (p = 0.0005), IGT without IFG (p = 0.0007), and IGT with IFG (p = 0.00009) groups compared with the NGT group	No	High
Sheikh et al. (25)	Single-center retrospective study. Follow-up: 5 years.	80 CF children aged 12 years (IQR 9.0-14.5). Glucose tolerance categories at baseline: INDET (8.7%), 1-h plasma glucose > 160 mg/dl (35%), NGT (56.3%)	INDET was associated with a significantly increased risk for future CFRD compared with no-INDET (OR 2.81, 1.43–5.51)	No	High
Schiaffini et al. (26)	Single-center prospective study. Follow-up: 2-5 years.	17 CF patients + 14 healthy controls tested with OGTT and CGM. Glucose tolerance categories at baseline: NGT (58%), IFG (5.9%), IGT (17.6%), IGT + INDET (11.8%), CFRD (5.9%)	Patients with PG1>160 mg/dl at baseline had 4 times more risk of developing CFRD; patients with PG1>200 mg/dl at baseline had 10 times more risk of developing CFRD	No	Moderate

**Table 7:** Diagnosis of diabetes and prediabetes in children with Cystic Fibrosis under 10 years of age.

References	Study design Follow up duration	Participants	Main outcomes	Rating upgrade/downgrade	Evidence level
Yi et al. (27)	Multicenter prospective study. Follow-up: unavailable	23 CF children aged 3 months to 5 years and 11 healthy control subjects tested with OGTT	All control subjects were NGT, while 39% of CF children had AGT status (2 CFRD, 2 INDET and 6 IGT). AUC of glucose was significantly higher in subjects with CF than control subjects (p=0.02)	No	Moderate
Fattorusso et al. (28)	Case report. Follow-up: 16 years	A CF patient diagnosed with GMD at 1 year with a very long-term follow-up.	This case report confirms the importance of paying attention to early GMDs in very young CF patients and seems to suggest that earlier therapy could ameliorate CF natural history	No	Low
Larson Ode K et al., 2010	Single-center retrospective study. Follow-up: 5 years.	62 CF children aged 6-9 years tested with OGTT. Glucose tolerance categories: AGT (50%) and NGT (50%)	10 years after study onset, 42% of AGT patients developed diabetes vs 3% of NGT patients. Age of CFRD onset was 12 ± 1 years in boys and 11 ± 1 years in girls	No	Moderate
Prentice et al. (29)	Single-center, observational, case-control study. Follow-up: unavailable	18 CF patients aged 0·9–5.5 years and 4 control subjects tested with CGM for 3 days	Peak SG was >11.1 mmol/L in 39% of CF patients. SG levels before age 6 years are associated with increased pulmonary inflammation and Pseudomonas aeruginosa infection	Downgrade (imprecision)	Moderate
Prentice et al. (30, 31)	Single-center observational study. Follow-up: unavailable	27 CF patients aged <10 years tested with OGTT. OGTT results were performed with results from CGM performed in 11 participants	There was a significant inverse correlation between weight and height z-scores with BG max (both p=0.02). AUC total was inversely correlated with weight, height and BMI z-score (p=0.01, p=0.009, p=0.02 respectively). A significant inverse correlation was also identified between fasting insulin level and elevated glucose on CGM, defined as AUC >7.8 mmol/L (p=0.027) or as % time > 7.8 (p=0.011)	No	High
Mozzillo et al. (32)	Single-center observational study. Follow-up: unavailable	152 CF children aged 2.4-18 years tested with OGTT Age groups: <6 years (n° 24), 6-10 years (n° 42), and >10 years (n° 86)	Prevalence of GMDs among three age groups were: between 2.4 and 5.9 years (n° 24), between 6 and 9.9 years (n° 42), and >10 years (n° 86)	No	High
Prentice et al. (30, 31)	Single-center observational study. Follow-up: 24 months	11 CF children (mean age 3.8 ± 2.5 years) tested with 3-day CGM at baseline, 12 months, and 24 months.	Three of the participants (27%) had normal CGM at all time-points. Seven children (64%) had a peak SG ≥11.1 mmol/L. None of the children had a peak SG ≥11.1 at every time point. Only four of the subjects (36%) did not have a peak SG ≥11.1 mmol/L at any time-point. Eight children (73%) spent more than 4.5% of their total time in the impaired range (> 7.8 mmol/L) at any time-point, and 5 (63%) had elevated percent time on more than one test	Downgrade (imprecision)	Moderate

**Table 8:** Effectiveness of therapy on glycemic control (HbA1c, fasting and 2 h post-meal serum blood sugar values).

References	Study design Follow-up duration	Participants	Treatment	Main outcomes	Rating upgrade/downgrade	Evidence level
Rolon et al. (33)	Retrospective-prospective case-control F/up: 5 years retrospective plus 5 years prospectively	14 CFRD patients age at T0: 15.3 y (range: 9 y 10 months to 21 y) compared with 14 non-diabetic CF patients	10 patients on two daily injections of a mixture of short- and intermediate-acting insulin 1 on basal-bolus regimen with short-acting insulin 1 on pre meal bolus-only 2 died shortly after insulin start	**HbA1c mean in cases** T0: no differences between cases and controls. T1 (n = 12) = 6.59 T2 (n = 8) = 7.37 T3 (n = 7) = 8.08 T4 (n = 7) = 7.51 T5 (n = 7) = 7.84	Downgrade (imprecision)	Low
Kolouskova et al. (34)	Prospective case–control F/up: 3 years	28 CF Insulinopenic patients 17 CFRD FH- and 11 IGT compared with 28 OGTT normal patients matched by sex, age and DOB	NPH insulin, once a day, 0.12 IU/kg (mean; range 0.09 – 0.25) before breakfast	**HbA1c %** T1 year: 5.5 vs 4.7 (p < 0,01) T2 Years: 5.3 vs 5.2 (N.S.) T3 Years: 6.4 vs 5.2 (p < 0,01)	Downgrade (imprecision, risk of bias)	Moderate
Grover et al. (35)	Randomized, cross-over F/up 12 weeks	19 CFRD with FH adults	12 week therapy with bedtime NPH vs 12 weeks of bedtime Glargine Boluses (Aspart) at least 3 times/day according to I:CHO of each patient	**HbA1c % (delta)** Glargine 6.4 ± 0.2 (-0.2 ± 0.2 from baseline) vs NPH 6.6 ± 0.2 (-0.2 ± 0.2 from baseline) (p 0,96) **Fasting Blood glucose (mg/dl)** Glargine 123 ± 4 (-8 ± 2, compared to baseline) vs NPH 125 ± 5 (0 ± 2, compared to baseline) (p 0,03) **2 H Post prandial glucose (mg/dl)** Glargine 150 ± 4 (+1.2± 0.5, compared to baseline) vs NPH 155 ± 9 (0.2 ± 0.5 compared to baseline) (p 0,85)	No	High
Frost et al. (36)	Prospective cohort study 2016-17 F/up: 12 months	52 Adults with CFRD diagnosed by CGM	15 patients: dietary modification 35 patients: on Detemir once daily (average initial dose 4.9 U) 2 patients on Lispro 2 U	**HbA1c** At baseline 39.4 mmol/mol At 3 months 31/37 HbA1c had <48 mmol/mol 20/32 had HbA1<40 mmol/mol	Downgrade (imprecision, risk of bias)	Low
Mozzillo et al. (37)	Prospective cohort study F/up: 12 months	22 CF patients: 4 AGT-CGMS 9 IGT 7 DM FH- 2 DM FH+ Mean age 12.4 ± 4.2 yr (range 2.6–19)	Glargine 1 daily before breakfast initial dose: 0.20 U/kg adjusted to obtain glycemia 70 - 140 mg/dl	**HbA1c** (average on 4 values in the last year before starting therapy vs 1 year on therapy) baseline 6.1 ± 0.1 (min 5.2 max 8.4) vs 6.3 ± 0.3 (min 5.5 max 11.6)	No	High
Minicucci et al. (38)	Multicenter, controlled, two-arm, randomized clinical study F/up: 18 months	34 CF patients with IGT (18 in the Glargine arm and 16 in the control arm). Median age 20 (range 11- 53)	Once daily Glargine up to a dosage of 0.15 U/kg/day, vs ordinary therapy (no hypoglycemic pharmacological treatment)	**HbA1c % difference** 0 - 18 months: −0.11 (−0.80; 0.30) vs 0.26 (−0.66; 0.95) p 0.04	No	Moderate
Moran et al. (39)	Multicenter, double blinded, comparative, randomized trial F/up: 12 months prospectively, plus 12 months retrospectively. (Dec 2007-2009)	74 adults with CFRD FH- and 26 with severe IGT at OGTT	Three arms of randomization: - Insulin Aspart (0.5 unit/15 g of CHO) - Repaglinide 2.0 mg orally - Oral placebo three times a day	+12 months **Fasting glucose**: no differences between groups **90-min postprandial glucose** CFRD FH-: significantly lower in those treated with Aspart vs placebo (p 0.06) 116 ± 4 mg/dl in the insulin group 138 ± 12 mg/dl in the placebo group 131 ± 7 mg/dl in the repaglinide group IGT: significantly lower in those treated with Aspart vs repaglinide (p 0.03) 114 ± 3 mg/dl in the insulin group 122 ± 4 mg/dl in the placebo group and 131± 9 mg/dl in the repaglinide group	Downgrade (imprecision)	High
Ballmann et al. (40)	Multicenter, open-label, comparative, randomized trial Dec 2009-2011 F/up: 24 months	75 patients >10 years, diagnosis of CFRD based on two consecutive OGTT in 6 months	34 patients on Repaglinide starting dose 0.5 mg ×3/die 41 patients on Insulin Regular (Actrapid) starting dose 0,05 UI/Kg/dose	**Fasting Glucose** No differences between the two groups **HbA1c** No difference between the two groups at baseline or after 12 or 24 months with repaglinide: 0.2% ± 0.7 with insulin vs −0.2% ± 1.3 (p 0.15)	Downgrade (risk of bias)	High
Hardin et al. (41)	Prospective cohort study F/up: 6 months	9 adults CFRD FH+	5 pts on D-Tron Plus pump 4 pts on Medronic paradigm 520 pump Therapy adjusted to maintain the following target -first morning: 95–120 mg/dl -pre-meal 75–110 mg/dl -postprandial: < 150 mg/dl.	**HbA1c** baseline 8.2% ± 1.9 vs 6 months after CSII 7.1%± 1.5 (p 0.05) **Fasting Blood glucose** (mg/dl) baseline 141 ± 41 vs 6 months after CSII 111± 27 (p 0.04) **2 H Post prandial glucose** (mg/dl) baseline 184± 44 vs 6 months after CSII 158± 32 (p 0.04)	Downgrade (imprecision)	Moderate
Geyer et al. (42)	Randomized, double-blind, crossover design Exenatide vs Placebo	Six patients, 10-25 years, 3 M 3 F, with CF and IGT	8.00-9.00 pm after at least 10 h long fasting. 48 h interval between the two drugs (exenatide/Placebo), ensuring complete clearance of exenatide. Intervention (Exenatide) in a dose of 2.5 micrograms 15 min prior to commencement of the test meal (standard meal)	**Postprandial blood glucose** Area under the curve over 240 min (AUC240) Exenatide 1431 ± 54 vs Placebo 1814 ± 109 mmol/L/min (p<0.0001) **Glucose peak postprandial value** Exenatide 7.65 ± 0.34 vs Placebo 9.53 ± 0.63 mmol/L (p<0.0001) Significant reduction of the glycemic response	Downgrade (imprecision)	Low
Gnanapragasam et al. (43)	Case report F/up: 3 months	A 21 year old CFRD patient	Semaglutide 0.13-0.16 mg weekly replaced prandial insulin Lispro in combination with Glargine 15 U	-Reduction in HbA1c from 9.1% to 6.7% -CGM: stable euglycemic pattern on CGM (TIR 68-77%; mean glucose, 142-163 mg/dl; SD, 51-65) during f/up -patient lost 2 Kg over the treatment period	No	Low

**Table 9:** Effectiveness of therapy on pulmonary function (eg forced expiratory volume (FEV1) and forced vital capacity (FVC).

References	Study design Follow-up period	Participants	Treatment	Main outcomes	Rating upgrade/downgrade	Evidence level
Rolon et al., (33)	Retrospective-prospective case-control F/up: 5 years retrospective plus 5 years prospectively	14 CFRD patients age at T0: 15.3 y (range: 9 y 10 mo to 21 y) compared with 14 non-diabetic CF patients	10 on two daily injections of a mixture of short- and intermediate-acting insulin 1 on basal-bolus regimen with short-acting insulin 1 on pre meal bolus-only 2 died shortly after insulin start	**FVC and FEV1** T-5 to T-1: lower in the cases (ns) -6 months: FVC 52 ± 20% vs 79 ± 20% (p= 0.01) FEV1 37 ± 19% vs 72 ± 23% (p = 0.01) FVC and FEV1 improved in the cases after the start of insulin therapy. Rate of FVC decline demonstrated in 5 of 7 patients after 5 y of insulin therapy (p = 0.1) Symptomatic cases seemed to benefit more than the screened cases	Downgrade (imprecision)	Low
Kolouskova et al., (34)	Prospective case–control F/up: 3 years	28 CF Insulinopenic patients, 17 DM FH - and 11 IGT) compared with 28 OGTT normal patients matched by sex, age and DOB	NPH insulin, once a day, 0.12 IU/kg (mean; range 0.09 – 0.25) before breakfast	**FEV1%** After 3 years FEV1 was lower in the untreated group compared to insulin treated patients who showed stable FEV 1 during insulin administration (61.0 ± 4.0 vs 73.5 ± 4.4; p 0.03)	Downgrade (imprecision, risk of bias)	Moderate
Frost et al. (36)	Prospective cohort study 2016-17 F/up: 12 months	52 Adult with CFRD diagnosed by CGM	15 patients on dietary modification 35 on Detemir once daily (average initial dose 4.9 U) 2 pz on Lispro 2 U	**FEV1** at 3 months +4.27% (1.1–7.48) p 0.01 worsened at 12 months (+1.07% from baseline, p 0.27)	Downgrade (imprecision, risk of bias)	Moderate
Hameed et al. (44)	Prospective cohort study F/up: median treatment of 0.8 years (range 1.3–2.2 years)	18 pts (7.2–18.1 yrs): 6 patients with CFRD 12 patients with early insulin deficiency (CFID1 and CFID2)	Detemir x 1/day starting from 0.1 unit/kg then adjusted for a blood glucose target of 4–8 mmol/l (72 -144 mg/dl) Median dose 0.13 units/kg/day median treatment duration 0.8 years (IQR 1.03)	**Delta %FEV 1** Insulin deficiency: -9.8% ± 9.3 vs +5.3 ± 11.5%, p=0.004 CFRD: +0.3 ± 8.3% vs –4.3 ± 18.6%, p=0.56 Whole Group: -7.9% ± 2.8 vs + 5.8 ± 13.4%, p=0.024 **Delta %FVC** Insulin deficiency: -6.8% ± 10.3 vs +5.8 ± 13.4%, p=0.024 CFRD: +4.0 ± 12.5% vs –3.8 ± 21.2%, p=0.34 Whole Group: -5.8% ± 14.3 vs +5.2 ± 12.7%, p=0.013	Downgrade (imprecision, risk of bias)	Moderate
Minicucci et al. (38)	Multicenter, controlled, two-arm, randomized clinical study F/up: 18 months	34 IGT adults with at least one of: 1. BMI <10th pc 2. loss of 1 pc class of BMI 3. FEV1 ≤80% than predicted; 4. FEV1 decrease ≥10%	Once daily Glargine up to a dosage of 0.15 U/kg/day, vs ordinary therapy (no hypoglycemic pharmacological treatment)	**FEV1%** There were no significant differences in FEV1 values between the two groups nor within groups.	No	Moderate
Mozzillo et al. (37)	Prospective cohort study F/up: 12 months	22 CF patients: 4 AGT-CGMS 9 IGT 7 CFRD FH- 2 CFRD FH+ Mean age 12.4 ± 4.2 yr (range 2.6–19)	Glargine x 1/day before breakfast initial dose: 0.20 U/kg adjusted to obtain glycemia 70 -140 mg/dl	**FEV1** (% of predicted for age sex race weight height), after 12 month therapy vs baseline 68.2 ± 6.2 (24.0/117.0) vs 77.1 ± 6.4 (37.0/118.0) p 0.01.	No	High
Fattorusso et al. (28)	Case report F/up: 16 years	Female CF patient. Intermittent diabetes during early childhood, IGT at 10 years, CFRD FH+ at 13 years.	0–9 y CFRD, intermittent requirement of rapid insulin 10 y IGT: Glargine 1/day (0.35 U/kg/day) 13 y CFRD-FH+ Rapid insulin + glargine (0.9 U/kg/day)	**FEV1%** At the age of 10 years: 97 13y: 97 16 yrs: 70.5 Earlier Glargine administration could have reduced the worsening of pulmonary function	No	Low
Moran et al. (39)	Multicenter, double blinded, comparative, randomized trial F/up: 12 months prospectively, plus 12 months retrospectively. (Dec 2007-2009)	74 adults with CFRD FH- and 26 with severe IGT at OGTT	Three arms of randomization: -Insulin Aspart (0.5 unit/15 g CHO) -Repaglinide 2.0 mg orally -Oral placebo three times a day	**FVC** CFRD FH group Insulin:-0.5 ± 2.0 (p 0.21) Repaglinide: -2.1 ± 2.2 (p 0.25) Placebo: -1.1 ± 2.5 (p 0.37) IGT group Insulin: -10.3± 4.2 (p 0.05) Repaglinide: -3.1± 5.6 (p 0.96) Placebo: -5.1± 3.7 (p 0.6) **FEV1 + 12 months** less decline in FEV1 in the insulin and repaglinide arms, but ns CFRD FH group Insulin:-1.8 ± 2.2 (p 0.21) Repaglinide: -1.3 ± 2.2 (p 0.1) Placebo: -3 ± 2.7 (p 0.5) IGT group Insulin: 12.1± 5.6 (p 0.12) Repaglinide: -4.9± 7.4 (p 0.82) Placebo: -11.5 ± 4.9 (p 0.05)	Downgrade (imprecision)	High
Ballmann et al. (40)	Multicenter, open-label, comparative, randomized trial F/up: 24 months (Dec 2009-2011)	75 patients >10 yrs. Diagnosis CFRD based on two consecutive OGTT in 6 months	34 patients on Repaglinide starting dose 0,5 mg x 3/day 41 on Regular Insulin (Actrapid) starting dose 0.05 UI/Kg/Dose	**FVC percentage of predicted** Change from baseline to 12 and 24 months: N.S. **FEV1** Change from baseline to 12 and 24 months: N.S.	Downgrade (risk of bias)	High

**Table 10:** Effectiveness of therapy on nutritional status [eg body mass index (BMI)].

References	Study design Follow-up duration	Participants	Treatment	Main outcomes	Rating upgrade/downgrade	Evidence level
Rolon et al. (33)	Retrospective-prospective case-control F/up: 5 years retrospective plus 5 years prospectively	14 CFRD patients age at T0: 15.3 y (range: 9 y 10 mo to 21 y) compared with 14 non-diabetic CF patients	10 on two daily injections of a mixture of short- and intermediate-acting insulin 1 on basal-bolus regimen with short-acting insulin 1 on pre meal bolus-only 2 died shortly after insulin start	**BMI z-score** Symptomatic cases had a decrease in their BMI z-score in the year preceding the onset of the insulin therapy (p = 0.03). BMI values increased significantly after the start of insulin therapy (p < 0.05).	Downgrade (imprecision)	Low
Kolouskova et al. (34)	Prospective case–control F/up: 3 years	28 CF 17 DM FH – and 11 IGT compared with 28 OGTT normal patients matched by sex, age and DOB Age at the diagnosis of CF: 0.1 – 13.3 years (median 3.6) Age at onset of the study: 11.2 – 21.6 years (median 15.4)	NPH insulin, once a day, 0.12 IU/kg (mean; range 0.09 – 0.25) before breakfast	**Weight z-score** NPH vs Controls T-1 Years: – 1.01 ± 0.13 vs – 1.03 ± 0.14 N.S. T0 – 1.08 ± 0.14 vs – 1.01 ± 0.13 N.S. T1 Years – 0.96 ± 0.14 vs – 0.98 ± 0.14 N.S. but significant increase from baseline in treated (p < 0.001) T3 Years – 0.83 ± 0.14 vs – 0.77 ± 0.16 N.S. but significant increase from baseline in treated (p < 0.001) **BMI z-score** NPH vs Controls T-1 Years – 0.78 ± 0.13 vs – 0.84 ± 0.13 N.S. T0 – 0.88 ± 0.14 vs – 0.77 ± 0.11 N.S. T1 Years – 0.75 ± 0.16 vs – 0.79 ± 0.13 N.S. but significant increase from baseline in treated (p < 0.05) T3 Years – 0.50 ± 0.20 vs – 0.62 ± 0.14 N.S. but significant increase from baseline in treated (p < 0.05) Weight and BMI significantly improved in the insulinopenic group following insulin administration	Downgrade (imprecision, risk of bias)	Moderate
Grover et al. (35)	Randomized, cross-over F/up 12 weeks	19 CFRD adults with FH	12 week therapy with bedtime NPH vs 12 weeks of bedtime Glargine Boluses (Aspart) at least 3 times/day according to I:CHO of each patient	**Weight (Kg)** Glargine 64.3 ± 2.4 (+1.2 ± 0.5 from baseline) vs NPH 65.7 ± 2.5 (+0.2 ± 0.5 from baseline) p 0.07 **Lean Body Mass (by DEXA) in Kg** Glargine 45.7 ± 1,9 (+0.3 ± 0.2 from baseline) vs NPH 45.7 ± 2 (+0.1 ± 0.2 from baseline) p 0.5 **Fat Body Mass (by DEXA) in Kg** Glargine 16.1 ± 1.4 (+0.7 ± 0.4 from baseline) vs NPH 16.7 ± 1.5 (+0.4 ± 0.4 from baseline) p 0.09	No	High
Hameed et al. (44)	Prospective cohort study F/up: median treatment of 0.8 years (range 1.3–2.2 years)	18 subjects (7.2–18.1 yrs): 6 patients with CFRD 12 patients with early insulin deficiency (CFID1 and CFID2)	Detemir x 1/day starting from 0.1 unit/kg then adjusted for a blood glucose target of 4–8 mmol/l (72 -144 mg/dl) Median dose 0.13 units/kg/day median treatment duration 0.8 years (IQR 1.03)	**Delta Weight z-score**: change pre insulin detemir vs post insulin detemir treatment Period of comparison pre therapy: 1 year in those who received insulin treatment for ≤1 year (n=10). Equivalent to duration of treatment in those who received insulin treatment for >1 year (range 1.3–2.2 years, n=8), Insulin deficiency (n = 12): -0.41 ± 0.43 vs + 0.22 ± 0.31 p = 0.003 CFRD (n = 6): –0.52 ± 0.25 vs +0.11 ± 0.24 p = 0.014 Whole Group: -0.45 vs ± 0.38 vs + 0.18 ± 0.29 p=0.0001 ΔWtSDS improved in 16 patients	Downgrade (imprecision, risk of bias)	Moderate
Frost et al. (36)	Prospective cohort study F/up: 12 months (2016-17)	52 Adult with CFRD diagnosed by CGM	15 pt dietary modification 35 pt on Detemir once daily (average initial dose 4.9 U) 2 pt on Lispro 2 U	**Weight (Kg)** In the insulin group at 3 months there was gain of weight (+ 1.23 kg, p 0.01); the improvement was not confirmed at 12 months. In the dietary modification group, no significant differences in weight after 3 and 12 months.	Downgrade (imprecision, risk of bias)	Moderate
Mozzillo et al. (37)	Prospective cohort study F/up: 12 months	22 CF patients: 4 AGT-CGMS 9 IGT 7 CFRD FH- 2 CFRD FH+ Mean age 12.4 ± 4.2 yr (range 2.6–19)	Glargine x 1/day before breakfast initial dose: 0.20 U/kg adjusted to obtain glycemia 70 -140 mg/dl	**BMI z score** (after 12 months of therapy vs baseline) Whole population: baseline 20.56 ± 0.26 (min -2.8 max 2.2) vs -0.37 ± 0.25 (min -2.9 max 2.2) N.S. After stratification for BMI z score ≤ -1 or > -1 The difference was significant (p 0.017) only in patients with BMI z-score ≤ -1.	No	High
Minicucci et al. (38)	Multicenter, controlled, two-arm, randomized clinical study F/up: 18 months	34 IGT adults with at least one of: 1. BMI <10th pc 2. loss of 1 pc class of BMI 3. FEV1 ≤80% than predicted; 4. FEV1 decrease ≥10%	Once daily Glargine up to a dosage of 0.15 U/kg/day, vs ordinary therapy (no hypoglycemic pharmacological treatment)	**BMI z-score** Glargine vs Controls At baseline: −0.45 (−3.06; 1.34) vs 0.05 (−1.46; 2.20) p 0.12 0 - 3 months: 0.00 (−0.72; 0.98) vs 0.05 (−0.34; 0.41) p 0.73 0 - 6 months: 0.00 (−1.40; 0.79) vs −0.11 (−1.67; 0.48) p 0.98 0 - 9 months: − 0.09 (−0.91; 0.59) vs −0.13 (−1.57; 0.54) p 1.00 0 - 12 months: −0.10 (−1.15; 1.19) vs −0.11 (−1.89; 1.27) p 0.98 0 - 15 months: −0.14 (−1.77; 1.50) vs −0.04 (−2.06; 0.61) p 0.87 0 – 18 months: −0.13 (−1.92; 1.44) vs 0.00 (−1.94; 0.78) p 0.97	No	Moderate
Fattorusso et al. (28)	Case report F/up: 16 years	Female CF patient. Intermittent diabetes during early childhood, IGT at 10 years, CFRD FH+ at 13 years.	0–9 y CFRD, intermittent requirement of rapid insulin 10y IGT: Glargine 1/day (0.35 U/kg/day) 13y CFRD-FH+ Rapid insulin + glargine (0.9 U/kg/day)	**BMI z-score** At the age of 10 years: -0.40 13y: - 0.18 16 yrs: - 1.52 Earlier Glargine administration could have reduced the worsening of nutritional status	No	Low
Moran et al. (39)	Multicenter, double blinded, comparative, randomized trial F/up: 12 months prospectively, plus 12 months retrospectively. (Dec 2007-2009)	74 adults with CFRD FH- and 26 with severe IGT at OGTT	Three arms of randomization: -Insulin Aspart (0.5 unit/15 g CHO) -Repaglinide 2.0 mg orally -Oral placebo three times a day	**BMI (kg/m^2^)** -12 - 0 months: BMI decline in all groups 0 + 12 months CFRD FH- group Insulin: 0.39 ± 0.21 (p 0.02) Repaglinide: 0.15 ± 0.21 (p 0.33) Placebo: -0.02 ± 0.25 (p 0.45) Patients who received repaglinide had an initial significant gain of 0.53 ± 0.19 BMI units within the first 6 months of therapy (p 0.01); not sustained at 12 months (p 0.33) IGT group Insulin: -0.42 ± 0.30 (p 0.45) Repaglinide: -0.71 ± 0.28 (p 0.45) Placebo: 0.24 ± 0.27 (p 0.02)	Downgrade (imprecision)	High
Ballmann et al. (40)	Multicenter, open-label, comparative, randomized trial F/up: 24 months (Dec 2009-2011)	75 patients >10 yrs. Diagnosis CFRD based on two consecutive OGTT in 6 months	34 patients on Repaglinide starting dose 0.5 mg x 3/day 41 on Regular insulin (Actrapid) starting dose 0,05 UI/Kg/dose	**BMI z-score** A significant change in BMI z-score was seen after 12 months Repaglinide −0.1 ± 0.4 Insulin +0,1 ± 0.4 p 0.02 but not after 24 months (N.S.)	Downgrade (imprecision)	High
Hardin et al. (41)	Prospective cohort study F/up: 6 months	9 adults CFRD FH+	5 pts on D-Tron Plus pump 4 pts on Medronic paradigm 520 pump Therapy adjusted to maintain the following target -first morning: 95–120 mg/dl -pre-meal 75–110 mg/dl -postprandial: < 150 mg/dl.	**Weight (Kg)** baseline 55.63 ± 3.5 vs 6 months after CSII 59.2 ± 3.3 p 0,01 **Lean Body Mass (Kg) by DEXA** baseline 48.2 ± 1.5 vs 6 months after CSII 50.6 ± 1.6 p 0.03	Downgrade (imprecision, publication bias)	Moderate

According to the ISPAD guidelines (5), the following two diagnostic categories of CFRD have been established for patients screened with oral glucose tolerance test (OGTT) during periods of stable CF clinical conditions, based on fasting and 2-h glucose values: CFRD without fasting hyperglycemia (CFRD-FH^-^) and CFRD with fasting hyperglycemia (CFRD-FH^+^) (**Table 11**). In addition, in symptomatic patients, the CFRD can be diagnosed if random blood glucose level is ≥200 mg/dL (≥11.1 mmol/L) on 2 or more occasions, and if HbA1c is ≥ 48 mmol/mol (6.5%) (48 mmol/mol), even though diagnosis of diabetes can also be made in CF patients that show HbA1c value below this range (45). During flare-up phases of the disease, when intravenous antibiotic therapy and/or systemic corticosteroid therapy is required, the diagnosis of CFRD can be made if a fasting glycemia ≥126 mg/dL (≥7 mmol/L) or a post-prandial blood glucose ≥200 mg/dL (≥11.1 mmol/L) is present for 48 h.

**Table 11:** Glucose Tolerance Categories in Cystic Fibrosis.

	Fasting Plasma Glucose (FPG)		2 h Plasma glucose value		Notes
	*mg/dl*	*mmol/l*	*mg/dl*	*mmol/l*	
**Normal glucose tolerance (NGT)**	< 100	< 5.6	< 140	< 7.8	All glucose levels < 140 mg/dl (7.8 mmol/L)
**Impaired fasting glucose (IFG)**	100 - 126	5.6 - 7	< 140	< 7.8	All glucose levels ≤ 140 mg/dl (7.8 mmol/L)
**Abnormal glucose tolerance 140 (AGT 140)**	< 100	< 5.6	< 140	< 7.8	Mid-OGTT glucose ≥ 140 mg/dl (7.8 mmol/L)
**Indeterminate glycemia (INDET)**	< 100	< 5.6	< 140	< 7.8	Mid-OGTT glucose ≥ 200 mg/dl (11.1 mmol/L)
**Impaired glucose tolerance (IGT)**	< 100	< 5.6	140 - 199	7.8 – 11	
**CFRD with fasting hyperglycemia**	≥ 126	≥ 7	≥ 200	≥ 11.1	
**CFRD without fasting hyperglycemia**	< 126	< 7	≥ 200	≥ 11.1

Due to the insidious onset of CFRD, once a year OGTT in all patients aged 10 and above is crucial for the diagnosis of CFRD and for the identification of high-risk subjects (5).

OGTT identifies patients with CFRD and with pre-diabetes using the following diagnostic categories: normal glucose tolerance (NGT); INDET; IGT (**Table 11**). Two more categories named AGT (4, 37) and impaired fasting glucose (IFG) can also to be considered (46) (**Table 11**).

### Impact of CFRD and Pre-Diabetes on CF Outcomes

Regarding the outcome “Impact of pre-diabetes on the clinical outcomes of CF”, 13 paper were included in this systematic review, 2 are multi-center and 11 are single-center studies; 8 were prospective observational studies while 5 were retrospective-observational studies. The number of enrolled patients ranged from 16 to 4,293 including both children and adults (**Table 5**).

#### Impact on Pulmonary Function

CF patients diagnosed with IGT or INDET had lower FEV1 and FVC levels compared to CF patients without glucose abnormalities (11–14) (evidence Moderate); glucose peaks >200 mg/dl (11.1 mmol/l) during a continuous glucose monitoring were associated with worse spirometry pulmonary function parameters (16) (evidence Low). Pulmonary function is not associated with alternative OGTT criteria (i.e. monophasic curve, glucose peak > 30 min, and/or 1 h ≥155 mg/dl) (15) (evidence Low).

Prediabetes was one of the most relevant predictors of deterioration of lung function defined as a significant decrease in FEV1 predicted value, during a 5 year-follow-up (17, 18) (evidence Moderate).

#### Impact on Growth and Nutritional Status

Auxological parameters, height-SDS and BMI-SDS, may be negatively influenced by prediabetes status in CF patients (11, 12, 14) (evidence Moderate).

Pediatric CF patients experienced a deterioration of their nutritional status and a negative impact on their final height due to prediabetes (14, 19) (evidence Moderate).

#### Impact on Pulmonary Exacerbations and Microbiological/Pathogens Colonization

A higher rate of pulmonary exacerbations, hospital admissions and outpatient clinic visits was observed in CF patients with CFRD and early glucose tolerance abnormalities compared to patients with NGT (11, 12, 19–21) (evidence Moderate).

CFRD and prediabetes diagnosis was recognized as independent risk factors for colonization by common CF pathogens, in particular for the acquisition of *Pseudomonas Aeruginosa*, its multiple antibiotic-resistant infection and its co-infection with other pathogens (11, 12, 19, 21–23) (evidence Moderate).

Regarding the outcome “Impact of prediabetes on the risk to develop CFRD”, 4 papers were included, 1 is a multi-center prospective study, 1 is a single-center prospective study and 2 are single-center retrospective studies. The sample size of the study populations ranged from 17 to 1,093 patients, especially children and adolescents (**Table 6**).

The results of these studies strongly support the evidence for an early detection of prediabetic conditions in CF patients, because IGT, IFG or INDET presented a five-years CFRD risk at least 10 times higher compared to CF patients with NGT (3, 24, 25). (evidence High). In addition, early glucose tolerance alterations defined by INDET during continuous glucose monitoring were also related to a higher risk of developing CFRD (25, 26) (evidence Moderate).

Regarding the outcome “Diagnosis of diabetes and prediabetes in patients with CF under 10 years of age**”** we included in this systematic review 7 papers. Among these, one is a multi-center prospective study, 4 are single-center prospective studies, one is a single-center retrospective study, and one is a case report. The number of enrolled patients ranged from 11 to 152 and age ranged from a few months-old to less than 10 years of age (**Table 7**). The analysis of these studies showed that in infants and young children with CF glucose derangements detected by OGTT are often diagnosed (3, 27–31) (evidence Moderate) and annual diabetes screening program in patients <10 years of age increased the early detection of CFRD (27, 28, 32) (evidence Low), leading to a more prompt and appropriate therapeutic approach (32) (evidence Very Low). Continuous glucose monitoring may be an alternative method for detecting glucose derangements in very young children with CF (29, 30) (evidence Moderate), particularly those with *Pseudomonas Aeruginosa* colonization (29) (evidence Moderate). Current evidence has also demonstrated that early diagnosis of prediabetes may be related to early clinical deterioration, particularly lung function and auxological parameters (29, 30) (evidence Moderate).

### Effectiveness of Therapy in CFRD

Regarding the outcome “Effectiveness on glycemic control”, we included in this systematic review 10 studies, 5 are randomized controlled trials (RCT), 3 are prospective cohort studies, one is a prospective case-control study, and one is a retrospective case-control study. The number of enrolled patients ranged from 9 to 100, from infancy to adulthood. Glycemic control has been analyzed in terms of glycated hemoglobin (HbA1c), fasting and 2 h post-meal blood glucose (**Table 8**). Different insulin and other drugs used are reported in **Table 8**.

Among the 3 studies on NPH insulin, in a retrospective - prospective case-control study, 10 pediatric patients with CFRD were treated with a mixture of short and intermediate acting insulin over a 10-year period, with no evidence of HbA1c improvement when compared to 14 non-diabetic matched controls (33) (evidence Low). In 28 adult patients, who were insulinopenic, diagnosed with IGT or CFRD- FH^-^, NPH insulin treatment (0.12–0.25 IU/kg/day) has been able to improve HbA1c during the first 2 years, but the effect vanished during the third year of treatment. NPH insulin did not increase the risk of hypoglycemia (34) (evidence Moderate*).* In an RCT, 19 adults with CFRD-FH^+^ were randomly assigned to treatment with NPH or with glargine while continuing pre-meal insulin aspart (at least 3 administration *per* day). After 12-week treatment, NPH and glargine had similar efficacy on metabolic control in terms of HbA1c and postprandial glycaemia, whereas in the group treated with glargine, FPG was significantly reduced. No severe hypoglycemia event occurred and the frequency of minor hypoglycemic episodes was not significantly different in the two groups (35) (evidence High).

Only one prospective cohort study used insulin detemir, showing that this insulin administered once a day has been able to maintain HbA1c value below 6.5% (48 mmol/mol) in adult patients with CFRD after 3 months of treatment. Moreover, 62.5% of patients had HbA1c values below 5.8% (40 mmol/mol), with an 8% decrease in time spent above 140 mg/dl (7.8 mmol/l) (36) (evidence Low).

Three studies evaluated the efficacy of basal insulin therapy using glargine, without any apparent effect of this insulin on HbA1c (**Table 8**). A significant decrease in HbA1c has been observed only after 18-month therapy in one study (38) (evidence Moderate).

Pre-meal insulin (aspart or regular Actrapid) was compared to repaglinide in two studies, with conflicting results (**Table 8**). A study in 81 adults with CF and FPG or IGT showed repaglinide more effective after 6-month follow-up but not after 12-month follow-up (39) (evidence High). In pediatric patients Actrapid insulin and repaglinide showed similar efficacy, with no severe hypoglycemia episodes in the 2 groups (40) (evidence High).

One study in 9 patients with CFRD-FH+ used insulin pump therapy for 6 months achieving a significant reduction of HbA1c, fasting and postprandial blood glucose, without severe hypoglycemia events (41) (evidence Moderate).

Two studies have been published on the use of GLP-1 receptor agonists (GLP-1 RA) in the treatment of CFRD (42, 43). In a double blind RCT study conducted on 6 adolescents and young adults with IGT, exenatide was administered for 48 h showing a significant reduction in postprandial blood glucose (42) (evidence Low); as well as Semaglutide administered weekly at a low dose, was able to replace prandial Lispro and control glycemia in combination with Glargine (43).

In regard to the outcome “Effectiveness on pulmonary function”, 9 studies were included in this systematic review, 3 are RCT, 3 are prospective cohort studies, and 3 are case-control studies, either prospective or retrospective. The number of enrolled patients ranged from 9 to 100, aged from less than 1 year to adulthood. The following therapies were tested in these studies, intermediate/NPH insulin (2 studies), insulin detemir (2 studies), insulin glargine (3 studies), rapid analogue insulin (2 studies), rapid insulin (1 study), repaglinide (2 studies), with conflicting results as shown in **Table 9**.

In a few studies, especially in pediatrics, it seems that insulin therapy has beneficial effects either on FVC or FEV1 (33) (evidence Low), while in adult patients the efficacy of insulin therapy seems to disappear after some time (34) (evidence Moderate*).* Similar data have been observed in other studies using insulin analogs either in adults (37) (evidence High) (36, 38) (evidence Moderate), or in pediatrics (44) (evidence Moderate) (28) (evidence Low).

Insulin aspart treatment compared to repaglinide or placebo, gave significant changes in the annual rate of decline in FVC, but not in FEV1 and in the number of acute exacerbations in subjects with Fc and IGT (39) (evidence High), while others observed that insulin Actrapid did not have any impact (40) (evidence High), or just limited one (28) (evidence Low) on pulmonary function decline.

Regarding the outcome “Effectiveness on nutritional status”, 11 papers were included in this systematic review, four are RCT, four are prospective cohort studies, one is a prospective case-control, and one is a case-control study. The number of enrolled patients ranged from 9 to 100, aged from a few months to adult age (**Table 10**).

Details about the relationship between the therapies used and the nutritional status of the patients with CF are given in **Table 10**. Summarizing, there is Low evidence that NPH insulin allows a significant improvement in BMI in children (33), but not in adults (34) (evidence Moderate), even if data are conflicting (35) (evidence Low). Better results have been found using insulin analogues, such as detemir either in pediatric patients (44) (evidence Moderate), or in adults (36) (evidence Moderate).

No significant differences in BMI-SDS were observed either in adolescents treated with Repaglinide compared to those treated with insulin Actrapid (40) (evidence High), or in adults (39) (evidence Moderate). Insulin pump therapy seemed to be effective in increasing weight and lean mass (41) (evidence Moderate).

## Discussion

This systematic review of the literature and the GRADE approach (8, 9) were able to demonstrate how much CFRD impacts on the clinical course of CF in terms of pulmonary function, pulmonary exacerbations, pulmonary microbiological colonization and auxological parameters.

Moreover, prediabetes emerged as an important predictor factor of either CFRD or worse prognosis of CF outcomes in both pediatric and adult patients. Evidence of prediabetes in children under 10 years of age is not unusual and is once again associated with a significant risk of progression to CFRD and worse clinical course of CF. A significant decline in pulmonary function was in fact observed in pediatric patients with prediabetes (14), as well as, in adult patients with prediabetes compared to patients with NGT (11–14).

Among prediabetes, IGT represents one of the most relevant predictors of the decline in pulmonary function (17) (18) and both CFRD and IGT have been recognized as independent risk factors for pulmonary exacerbations and colonization by common CF pathogens, in particular by *Pseudomonas Aeruginosa*, and its form of infection characterized by multi-resistance to antibiotic therapy and co-infection with other pathogens (11, 16, 19, 21–23, 25).

Glycemic peaks higher than 200 mg/dl (11.1 mmol/l) were recorded with continuous glucose monitors in patients screened with OGTT, with normal fasting and 2-h blood glucose, and these glycemic peak were related to a worsening of spirometry parameters (16). A very recent study, not found by the search for the systematic review that was updated till October 2020, analyzed the correlation between intermittent scan CGM (isCGM) and OGTT data in a cohort of 32 children with CF. The isCGM percent of measurements >140 mg/dL (7.8 mmol/L) and the number of peaks per day >200 mg/dL (11 mmol/L) have correlations with intermediate OGTT glucose time points, but not the 2-h glucose value. Moreover patients with abnormal glucose tolerance (AGT) had lower lung function than those with normal glucose tolerance demonstrated by both FEV1% predicted and lung clearance index (47).

Among patients with CF, auxological parameters are significantly impaired in patients with CFRD than patients with NGT, also before diabetes diagnosis (14) and can be negatively influenced by prediabetes (11, 12, 44).

Evaluating the impact of different therapeutic strategies on glycemic control, pulmonary function and auxological parameters in patients with CFRD, glargine seems to be the most studied insulin. Data showed with moderate to high level of evidence that glargine therapy led to a significant improvement in HbA1c, the gold standard metric for long-term glycemic control, only in adult IGT patients (38), but not in children (37). In addition, in CFRD-FH^+^ adult patients glargine had the same efficacy than NPH insulin (35). Data about detemir are more confusing (36, 44).

A significant improvement in HbA1c value was demonstrated in CFRD patients treated with insulin pump therapy (41).

The analysis of the efficacy of pre-meal insulin or oral antidiabetic agents in controlling postprandial hyperglycemia in CFRD showed that aspart insulin is more effective than repaglinide (39), and regular insulin is as effective as repaglinide in patients with HbA1c <7% (40).

Glargine insulin in CFRD patients did not increase the risk of hypoglycemia when compared to NPH (35), and repaglinide does not increase the number of hypoglycemic events compared to regular insulin (40).

In both, adult and pediatric patients with CFRD or prediabetes, insulin therapy with NPH (32), as well as with Detemir (44) and glargine (37, 38) preserved pulmonary function and reduced the number of respiratory exacerbations, whereas therapy with regular human insulin or rapid analogue insulin or repaglinide had no impact on pulmonary function (28, 39, 40).

Regarding auxological and nutritional status parameters, in adults with diabetes or prediabetes, NPH insulin preserved the decay of BMI-SDS (34). Insulin detemir in pediatric patients with CFRD improved BMI-SDS (44), while this effect was not sustained in adult patients 37. In pediatric subjects diagnosed with AGT, IGT or CFRD, glargine insulin improved the BMI-SDS only in patients with poor nutritional status (BMI SDS <−1) (37, 38). In adults, insulin therapy with rapid analogue increased BMI in those diagnosed with IGT, but not in those with CFRD FH^+^ (39), while insulin pump therapy produces an increase in weight and lean mass measured with DEXA after 6 months of therapy (41). Repaglinide and regular insulin in CFRD patients have no impact on BMI-SDS (40).

## Limitations

To obtain the best evidence to answer our research questions, we have made a great effort to collect studies by establishing strict inclusion criteria. Even if low-quality studies have been excluded, a few limitations still exist, that should be acknowledged for the evaluation and interpretation of the results, and consequently, the recommendation summarized in this review: 1) few RCT studies have been found, particularly for the outcome related to “effectiveness of treatment”; 2) only a few studies had sample sizes larger than 100 patients, and 3) continuous glucose monitoring was used in a limited number of studies.

## Conclusions and Recommendations for The Clinical Practice

CF is a complex multi-organ disease requiring a comprehensive multidisciplinary treatment program. CFRD deserves special knowledge and expertise to be adequately diagnosed and treated. For these reasons, after a systematic review of the literature, the Diabetes Study Group of the ISPED has agreed to provide practical recommendations based on both evidence from current literature and clinical experience.

1. In patients with CF, OGTT is the gold-standard method for the screening of glucose abnormalities during periods of clinical stability (absence of respiratory exacerbation and/or use of antibiotic and/or steroid therapy, and in case of organ transplantation). Patients should be tested once a year -**Strong in favor-**.
2. Random BG and fasting BG measurement are recommended during periods of pulmonary exacerbations and/or use of glucocorticoid therapy, enteral nutrition and in case of diabetes symptoms -**Strong in favor-**.
3. Current evidence did not support the use of continuous glucose monitoring as a diagnostic and/or screening tool; however, it is considered very useful for monitoring subjects with IFG, IGT or INDET and during high risk conditions (i.e.: pulmonary exacerbations and/or use of steroids) - **Strong in favor-**.
4. Subjects with prediabetes are at risk of developing CFRD, thus close monitoring of glucose metabolism with OGTT and 2-week CGM at 6-month intervals is recommended **-Conditional in favor-**.
5. In children <10 years of age, starting from at least 6 years of age or even earlier if possible, it is advisable to screen for alterations of glucose metabolism at least once a year, whenever possible, and if IGT or INDET conditions are diagnosed a close monitoring of BG levels is recommended (OGTT at 6-month intervals; continuous glucose monitoring as a possible adjunctive tool for glucose monitoring during respiratory exacerbations and/or use of steroids), **-Conditional in Favor-**.
6. Treatment with a basal analogue (glargine) should be started in patients affected by CFRD FH^+^ at a dosage of 0.2 IU/kg/day **-Conditional in Favor-**.
7. Postprandial hyperglycemia should be treated with rapid analogue at initial dosage of 0.05 to 0.1 UI/kg before meal or an insulin/carbohydrate ratio starting with 1UI: 15 g and 1UI: 30 g ratio and subsequently modified on the basis of different intakes of the day and of the degree of insulin resistance at each moment. **-Conditional in favor-**.
8. The risk of hypoglycemia due to insulin therapy is low**, -Strong in favor-.** Treatment of CFRD with insulin could result in an improvement in fasting and postprandial blood sugar levels more than in the HbA1c value, **-Strong in favor-**.
9. Insulin pump therapy, although rarely used in these patients, is a valid and effective alternative to multiple daily injections **-Strong in favor-**.
10. In patients diagnosed with IGT, if clinically compromised, treatment with basal insulin analogue is recommended starting with 0.1 to 0.2 UI/kg/day with subsequent adjustments based on the glycemic trend, -**Conditional in Favor**-.
11. In patients diagnosed with other prediabetic conditions, in particular INDET and AGT, early initiation of insulin therapy could be beneficial and this possibility should be taken into account after a complete evaluation of the patient on the basis of the annual trend of the OGTT results, glucometrics, glycemic values measured during pulmonary exacerbations and/or steroid therapy, -**Conditional in Favor**-.
12. In all CF patients with diabetes and prediabetes treated with insulin analysis of glucose metrics with CGM may be useful for monitoring insulin treatment, -**Conditional in Favor**-.

## Collaborators

Collaborators of the Diabetes Study Group of the Italian Society for Pediatric Endocrinology and Diabetology (ISPED). A complete list of the members of the Diabetes Study Group of the ISPED can be found in the supplementary material online.

## Author Contributions

EM and RF engaged in literature retrieval of the articles, have analyzed the results, and wrote the manuscript. CP, SP, AC, and DP performed records screening and assessed eligibility, compilation of evidence and of evidence tables. AES reviewed the records screening and contributed to wrote the manuscript. GM, VCal, VCau, VCh, GD, AF, APF, FL, DP, MCM, EP, BP, IR, ST, and SZ discussed and commented on literature analysis. RS and CM critically revised the manuscript. All authors contributed to the article and approved the submitted version.

## Conflict of Interest

The authors declare that the research was conducted in the absence of any commercial or financial relationships that could be construed as a potential conflict of interest.
