# Supplementary material for: Diabetes and Prediabetes in Children With Cystic Fibrosis: A Systematic Review of the Literature and Recommendations of the Italian Society for Pediatric Endocrinology and Diabetes (ISPED)
Source: Front Endocrinol (Lausanne). 2021 Apr 29;12:673539. doi: 10.3389/fendo.2021.673539 (PMC8130616; doi:10.3389/fendo.2021.673539)
Supplement: Supplementary file 1 [file DataSheet_1.pdf]

DIABETES STUDY GROUP OF ISPED

NAME	CITY
ALOE MONICA	CATANZARO
AMADEO SIMONA	
ARNALDI CLAUDIA	VITERBO
BANIN PATRIZIA	FERRARA
BARBETTI FABRIZIO	ROMA
BASSI MARTA	GENOVA
BECCARIA LUCIANO	MILANO
BENELLI MARZIA	LECCE
BERIOLI MARIA GIULIA	PERUGIA
BERTELLI ENRICA	ALESSANDRIA
BIAGIONI MARTINA	ANCONA
BOBBIO ADRIANA	AOSTA
BOCCATO STEFANO	BELLUNO FELTRE
BOLOGNA ORIANA	TRAPANI
BONFANTI RICCARDO	MILANO
BONTEMPI FRANCO	MANTOVA
BONURA CLARA	MILANO
BRACCIOLINI GIULIA	ALESSANDRIA
BRUFANI CLAUDIA	TARQUINIA
BRUZZI PATRIZIA	MODENA
BUONO PIETRO	NAPOLI FEDERICO II
CADARIO FRANCESCO	ALESSANDRIA
CALCATERRA VALERIA	PAVIA
CAPPA MARCO	ROMA
CARDANI ROBERTA	VARESE
CARDELLA FRANCESCA	PALERMO
CARDINALE GIULIANA	CASARANO (LE)
CASERTANO ALBERTO	NAPOLI FEDERICO II
CASTIGLIONE CRISTINA MARIA	PALERMO
CAUVIN VITTORIA	TRENTO
CHERUBINI VALENTINO	ANCONA
CHIARELLI FRANCO	CHIETI
CHIARI GIOVANNI	PARMA
CIANFARANI STEFANO	ROMA
CICCHETTI MARIO	CAMPOBASSO
CIRILLO DANTE	LEGNANO
CITRINITI FELICE	CATANZARO
COCCIOLI SUSANNA	BRESCIA
COGLIARDI ANNA	LECCO

CONFETTO SANTINO	NAPOLI
CONTERAS GIOVANNA	VERONA
CORO’ ANNA	TREVISO
CORSINI ELISA	FIRENZE
COTELLESSA MARIO	IMPERIA
CRESTA NICOLETTA	CAMPOBASSO
CRILLO DANTE	MILANO
CRINO’ ANTONINO	ROMA
D’ANNUNZIO GIUSEPPE	GENOVA
DE BERARDINIS FIORELLA	CETRARO (CS)
DE DONNO VALERIA	CUNEO
DE FILIPPO GIANPAOLO	
DE MARCO ROSARIA	COSENZA
DE SANCTIS LUISA	TORINO
DELVECCHIO MAURIZIO	BARI
DEODATI ANNALISA	ROMA
FALESCHINI ELENA	TRIESTE
FATTORUSSO VALENTINA	NAPOLI
FAVALLI VALERIA	MILANO
FEDERICO GIOVANNI	PISA
FELAPPI BARBARA	BRESCIA
FERRITO LUCIA	ANCONA
FICHERA GRAZIELLA	SAVONA
FONTANA FRANCO	ALESSANDRIA
FORNARI ELENA	VERONA
FRANCESCHI ROBERTO	TRENTO
FRANCO FRANCESCA	UDINE
FRANZESE ADRIANA	NAPOLI FEDERICO II
FREZZA ELDA	BARI
FRONGIA ANNA PAOLA	CAGLIARI
FRONTINO GIULIO	MILANO
GAIERO ALBERTO	SAVONA
GALLO FRANCESCO	BRINDISI
GARGANTINI LUIGI	BERGAMO
GIANI ELISA	MILANO
GIORGETTI CHIARA	ANCONA
GIULIA BIANCHI	
GRAZIANI VANNA	RAVENNA
GUALTIERI ANTONELLA	AVEZZANO
GUASTI MONICA	FIRENZE
IAFUSCO DARIO	NAPOLI II UNIV
IANNICELLI GENNARO	SALERNO
IANNILLI ANTONIO	ANCONA
IGNACCOLO GIOVANNA	TORINO

INGLETTO DARIO	TRICASE (LE)
INNAURATO STEFANIA	VICENZA
INZAGHI ELENA	ROMA
IOVANE BRUNELLA	PARMA
IUGHETTI LORENZO	MODENA
KAUFMANN PETER	BOLZANO
LA LOGGIA ALFONSO	CALTANISSETTA
LAPOLLA ROSA	POTENZA
LASAGNI ANNA	REGGIO EMILIA
LAZZARO NICOLA	CROTONE
LENZI LORENZO	FIRENZE
LERA RICCARDO	ALESSANDRIA
LEVANTINI GABRIELLA	CHIETI
LOMBARDO FORTUNATO	MESSINA
LONERO ANTONELLA	BARI
LONGHI SILVIA	BOLZANO
LOPRESTI DONATELLA	CATANIA
LORINI RENATA	GENOVA
LUCCHESI SONIA	LIVORNO
LUCIA PAOLA GUERRAGGIO	TRADATE (VA)
LUCIERI SERGIO	CASTROVILLARI
MACELLARO PATRIZIA	LEGNANO
MAFFEIS CLAUDIO	VERONA
MAINETTI BENEDETTA	FORLI’
MALTONI GIULIO	BOLOGNA
MAMELI CHIARA	MILANO
MAMMI' FRANCESCO	LOCRI
MANCA BITTI MARIA LUISA	ROMA PTV
MANCO MELANIA	ROMA
MARINO MONICA	ANCONA
MARIANO MATTEO	FOGGIA
MARIGLIANO MARCO	VERONA
MARSCIANI ALBERTO	RIMINI
MASTRANGELO COSTANZO	FOGGIA
MATTEOLI MARIA CRISTINA	ROMA
MAZZALI ELENA	MANTOVA
MESCHI FRANCO	MILANO
MIGLIACCIO ANTONELLA	CATANZARO
MINUTO NICOLA	GENOVA
MONCIOTTI CARLA MARIA	PADOVA
MORANDI ANITA	VERONA
MORGANTI GIANFRANCO	BUSTO ARSIZIO (VA)
MOZZILLO ENZA	NAPOLI FEDERICO II
MUSOLINO GIANLUCA	VARESE

NUGNES ROSA	NAPOLI
ORTOLANI FEDERICA	BARI
PARDI DANIELA	MASSA CARRARA
PASCARELLA FILOMENA	CASERTA
PASQUINO BRUNO	BOLZANO
PASSANISI STEFANO	MESSINA
PATERA IPPOLITA PATRIZIA	ROMA
PEDINI ANNALISA	RIMINI
PENNATI CRISTINA	BERGAMO
PERROTTA ANGELO	CASERTA
PERUZZI SONIA	
PEVERELLI PAOLA	BELLUNO FELTRE
PEZZINO GIULIA	CATANIA
PICCINI BARBARA	FIRENZE
PINELLI LEONARDO	VERONA
PIONA CLAUDIA ANITA	VERONA
PIREDDA GAVINA	OLBIA
PISCOPO ALESSIA	NAPOLI II UNIV
PISTONE CARMELO	PAVIA
POCECCO MAURO	CESENA
PRANDI ELENA	BRESCIA
PREDIERI BARBARA	MODENA
PROCOLO DI BONITO LINO	NAPOLI II UNIV
PULCINA ANNA	FIRENZE
QUINCI MARIA	MAZARA DEL VALLO (TP)
RABBONE IVANA	NOVARA
RANDAZZO EMIOLI	PISA
RAPINI NOVELLA	ROMA
REINSTADLER PETRA	BOLZANO
RICCIARDI ROSSELLA	CAGLIARI
RIGAMONTI ANDREA	MILANO
RIPOLI CARLO	CAGLIARI
ROPPOLO ROSANNA	PALERMO
RUTIGLIANO IRENE	SAN GIOVANNI ROTONDO
SABBION ALBERTO	VERONA
SALARDI SILVANA	BOLOGNA
SALVATONI ALESSANDRO	VARESE
SAPORITI ANNA	VARESE
SARDI RITA	MASSA CARRARA
SAVASTIO SILVIA	NOVARA
SCANU MARIAPIERA	CARBONIA
SCARAMUZZA ANDREA	CREMONA
SCHIAFFINI RICCARDO	ROMA
SCHIVEN ELEONARDO	ARZIGNANO (VI)

SECCO ANDREA	ALESSANDRIA
SESSA LINDA	NAPOLI II UNIV
SOGNO VALIN PAOLA	IMOLA
SORDELLI SILVIA	MANTOVA
SPALLINO LUISA	CERNOBBIO
STAGI STEFANO	FIRENZE
STAMATI FILOMENA	COSENZA
SUPRANI TOSCA	CESENA
TALARICO VALENTINA	CATANZARO
TIMPANARO TIZIANA	CATANIA
TINTI DAVIDE	TORINO
TIRENDI ANTONELLA	MANTOVA
TOMASELLI LETIZIA	CATANIA
TONI SONIA	FIRENZE
TORNESE GIANLUCA	TRIESTE
TRADA MICHELA	TORINO
TRETTENE ADOLFO ANDREA	VARESE
TUMINI STEFANO	CHIETI
VALERIO GIULIANA	NAPOLI II UNIV
VANELLI MAURIZIO	PARMA
VENTRICI CLAUDIA	LOCRI
VISCARDI MATTEO	MILANO
ZAFFANI SILVANA	VERONA
ZAMPOLLI MARIA	COMO
ZANETTE GIORGIO	PORDENONE
ZANFARDINO ANGELA	NAPOLI II UNIV
ZECCHINO CLARA	BARI
ZEDDA MARIA ANTONIETTA	CAGLIARI
ZONCA SILVIA	
ZUCCHINI STEFANO	BOLOGNA
