# Supplementary material for: Diabetes and Prediabetes in Children With Cystic Fibrosis: A Systematic Review of the Literature and Recommendations of the Italian Society for Pediatric Endocrinology and Diabetes (ISPED)
Source: Front Endocrinol (Lausanne). 2021 Apr 29;12:673539. doi: 10.3389/fendo.2021.673539 (PMC8130616; doi:10.3389/fendo.2021.673539)
Supplement: Supplementary file 2 [file DataSheet_2.pdf]

PRISMA 2009 Flow Diagram

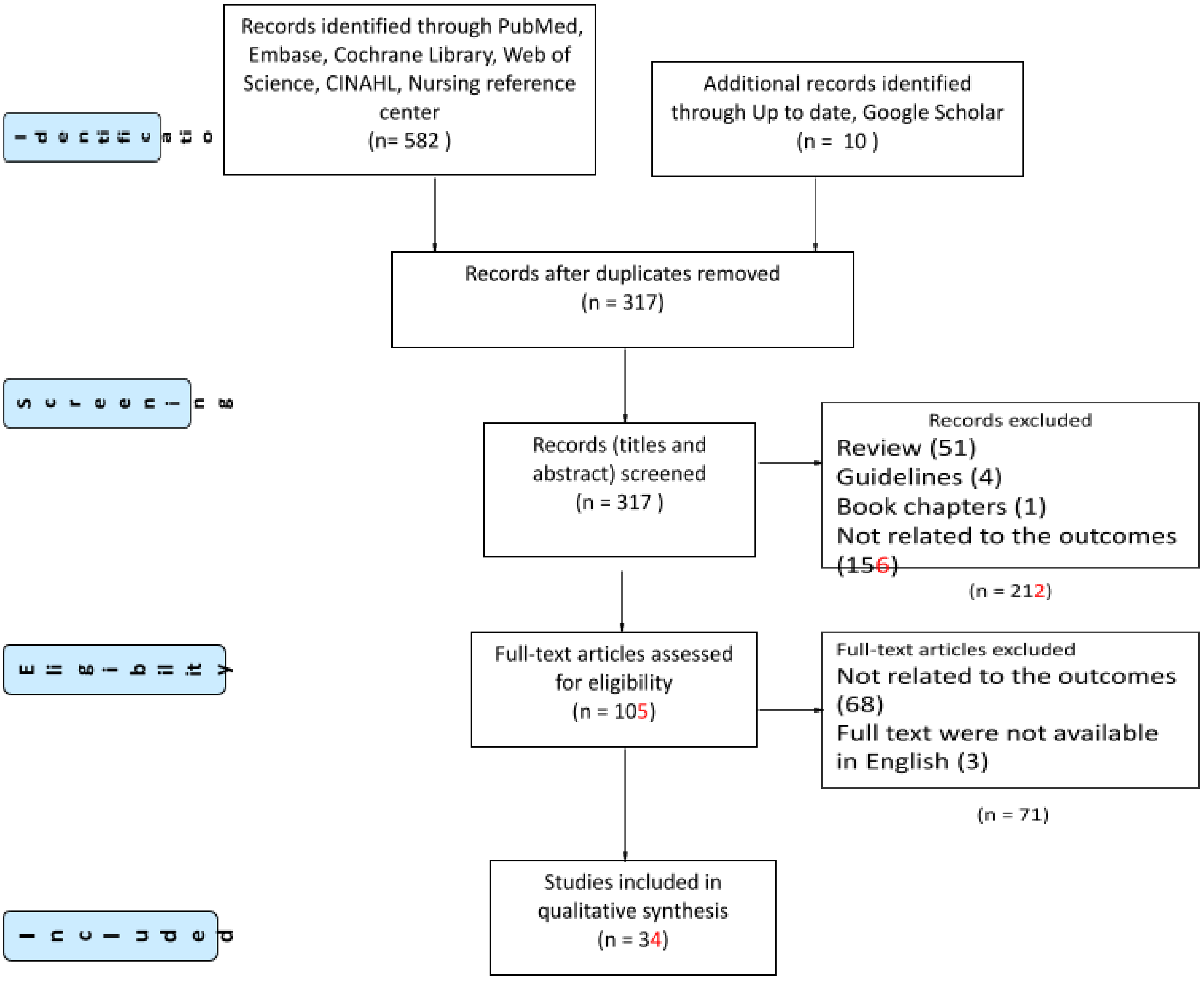

From: Moher D, Liberati A, Tetzlaff J, Altman DG, The PRISMA Group (2009). *P*referred *R*eporting *I*tems for *S*ystematic Reviews and *M*eta-*A*nalyses: The PRISMA Statement. PLoS Med 6(7): e1000097. doi:10.1371/journal.pmed1000097

For more information, visit www.prisma-statement.org.
